# Association of pro-inflammatory cytokine gene polymorphisms with schizophrenia in South Indian population

**DOI:** 10.1186/1755-8166-7-S1-P41

**Published:** 2014-01-21

**Authors:** Lekshmy Srinivas, NV Neetha, Chandrasekharan Nair, Priya M Allencherry, Moinak Banerjee

**Affiliations:** 1 Human Molecular Genetics Laboratory, Rajiv Gandhi Centre for Biotechnology, Trivandrum, Kerala, India; 2 Nair’s Hospital, Ernakulam, Kerala, India; 3 Mental Health Centre, Trivandrum, Kerala, India

## Background

Schizophrenia is a severe and debilitating mental illness. Around 0.26% of people in South India suffer from schizophrenia. It is a complex disorder which may involve multiple genes with mild to moderate effect and non-genetic risk factors like environmental and psychological assaults. Cytokines, regulators of immune/inflammatory reactions and brain development, emerge as part of a common pathway of genetic and environmental components of schizophrenia. Our study explored the association of polymorphisms in cytokine genes with schizophrenia, the interaction of these genes in the causation of the disease and the population genetics of cytokine gene polymorphisms.

## Materials and methods

We performed a case-control association study using 248 patients from Kerala, South India and 244 ethnically matched normal healthy controls. We screened polymorphisms in 10 cytokine genes (IL1A, IL1B, IL1RN, IL3, IL4, IL6, IL10, IFNG, TNFA and TGFB1). Genomic DNA was isolated from blood and genotyping was carried out by PCR-RFLP, TaqMan allelic discrimination and KASPar assays. Allelic, genotypic, haplotypic and diplotypic frequencies were calculated and compared. Gene-gene interactions among cytokine genes were also assessed.

## Results

We found significant association of SNPs in pro-inflammatory cytokine genes IL1A, IL6, TNFA and IFNG with schizophrenia in the Kerala population. No significant association was observed with any of the anti-inflammatory cytokine genes IL4, IL10 and TGFB1. Our study provides significant evidence for strong gene-gene interactions among pro-inflammatory cytokine genes in the development of schizophrenia. Our findings support the immune hypothesis in the predisposition to schizophrenia.

## Conclusions

The phylogenetic analysis of Kerala population with HapMap populations show proximity to HapMap Gujarati Indian population (GIH), Mexican population (MEX) and the African populations ASW, MKK and YRI. This similarity can be attributed similar selection pressures that lie in the same latitudinal belt indicating similar environmental conditions with respect to temperature, rainfall, pathogens, sunlight etc. This information might facilitate the identification of clinical subgroups of patients with strong immunological basis for the outcome of the disease.

